# Cheap Talk with Multiple Strategically Interacting Audiences: An Experimental Study

**DOI:** 10.1371/journal.pone.0163783

**Published:** 2016-10-03

**Authors:** Xinyu Li, Ronald Peeters

**Affiliations:** 1 Managerial Economics, Faculty of Business Administration and Economics, University of Paderborn, Paderborn, Germany; 2 Department of Economics, School of Business and Economics, Maastricht University, Maastricht, The Netherlands; Middlesex University, UNITED KINGDOM

## Abstract

We consider a cheap-talk setting that mimics the situation where an incumbent firm (the sender) is endowed with incentives to understate the true size of the market demand to two potential entrants (the receivers). Although our experimental data reveals that the senders’ messages convey truthful information and this is picked up by the receivers, this overcommunication (relative to standard theoretical prediction) does not enhance efficient entry levels (and payoffs) to beyond what can be achieved without communication. The reason is that receivers fail to optimally translate the information received in their entry decision, possibly due to overcautiousness.

## Introduction

There are many real-life situations in which some party possesses information while others do not. In some of these situations, the informed party voluntarily reveals a certain amount of possessed information at no cost in the hope of influencing the actions of the uninformed parties. This kind of costless, non-verifiable and non-binding communication is called cheap talk. The informed party strategically chooses which information to transmit and which not in order to induce the uninformed parties to take actions that it prefers. The choice of message becomes more complicated when the uninformed parties interact with each other.

Think of a traffic congestion problem. When drivers hear from a radio station that there is a two-hour traffic jam ahead, they naturally want to switch to an alternative road on which there is no congestion. However, if all drivers do so, there will be a traffic jam on the alternative road. The drivers need to coordinate on who should switch and who should stay in order to efficiently streamline the road usage. If some of the drivers are expected to stay and some are expected to switch when they hear the traffic jam is one-hour, the radio station could understate the true degree of congestion to the benefit of the efficiency of road usage. The radio station does not tell the truth even though it has no conflict of interest with the drivers. Things become more interesting when the informed party has an interest conflict with the uninformed parties.

Consider a dating platform announcing its number of subscribers. Given that potential new subscribers prefer platforms with more users, the platform has an incentive to overstate the current number of subscribers in order to attract more users. If these potential subscribers understand the platform’s incentive, no one can regard the announced number credible. Then, it does not matter for the platform which number to announce. However, if some potential users believe the inflated number and join the platform, it is also beneficial for those who don’t believe to join. Now it does matter for the platform which number to announce. Clearly, these examples illustrate that the complexity of the strategic relation between the informed party and the uninformed interacting parties may have an influence on the efficiency of the resulting outcome.

The literature offers several contributions that consider communication between one sender and two receivers in which the two firms do not act in strategic interdependence. In [1] it is shown that the presence of multiple audiences may induce public messages to become informative where messages would not carry any information in any isolated bilateral interaction, but can also complicate information revelation where this would happen in all isolated bilateral communications. All depends on the level of agreement between the sender and the receivers on which action is best to take. Their predictions are confirmed in [2] in a controlled laboratory experiment. In [3] it is also found that senders tend to be more sophisticated in their choices when increasing the number of receivers from one to two.

As already mentioned, these studies restrict attention to the situation where the payoffs of the receivers are independent of each other’s actions. In contrast, in our study, we assume strategic interdependence between the two receivers. This strategic interdependence may suppress the sender’s feeling of guilt from sending untruthful messages (deception) due to the responsibility for a receiver’s payoff being shared with the other receiver, and feelings of guilt are affected by the sharing of responsibility (cf. [4]). Moreover, strategic interdependence adds additional uncertainty to the receiver’s choice problem. Now the receiver is not only uncertain about the truthfulness of the sender’s message, but also about the action taken by the other receiver. Compared to the one-receiver setting (or multi-receiver without strategic interdependence), strategic interdependence between receivers may render receivers more cautious to take risky actions. This possibly more cautious behavior feeds back in the receiver’s expected costs and benefits of lying.

Also in [5] game is considered with two interacting receivers and one sender. The sender communicates a payoff relevant state of the world to the receivers who play a coordination game with multiple equilibria. They find that by sending a vague message in the experiment the sender can mask the existing payoff asymmetry and make receivers believe on average they are playing the equal payoff game where they can easily coordinate using the focal point. Notice that the receivers and the utilitarian benevolent sender agree that coordination is desirable. The alignment of the preferences between the receivers and the sender leaves more room to the sender to manoeuvre the message space over the states of the world and makes communication efficiency enhancing. However, if their preferences are not totally aligned, interesting questions are how the sender sends messages, how the receivers respond to the messages, and whether communication improves efficiency. We investigate these questions in the following experiment.

The cheap talk setting (treatment CT) that we consider in our experiment resembles the situation in which an incumbent firm can signal market conditions to two possible entrants via one public message. Next, upon receiving this message, independently of each other, the receivers decide whether to enter the market or not. The three possible market conditions refer to the demand in the market, which is either small, medium or large. There are guaranteed losses for potential entrants to enter a small demand market and there are guaranteed benefits for both to enter a large demand market. In case of a medium demand market, there is room for only one potential entrant to enter with positive benefit. Irrespective of the market condition, the incumbent firm has a preference to deter entry. So, regarding the alignment of preferences between the incumbent and the potential entrants, there is perfect alignment in a small demand market and they are perfect antagonists in the large demand market, while it is a bit mixed in the medium demand market. The incumbent can deter entry by making the potential entrants believing that the demand is small, in which case the dominant action for them is to not enter. As a result, messages become noncredible and only babbling equilibria exist (cf. [6]). In babbling equilibria, the public message does not contain any information about the true market condition and consequently does not influence the actions of the potential entrants.

Despite there only being babbling equilibria, from an ex ante perspective, all parties involved would prefer full revelation to any babbling equilibrium, since full revelation generates higher payoffs to all parties than babbling equilibria. Thus, there is a mutual benefit to ameliorate the failure to communicate. In order to assess whether cheap talk yields more efficient entry levels and higher payoffs, we implement two baseline treatments: one in which full information is given to the potential entrants (treatment FI) and one in which the communication channel is disabled (treatment NI). If the overcommunication (relative to sequential equilibrium assuming rational and egoistic agents) that is found in one-sender/one-receiver experiments (cf. [7–10]), is also found in our one-sender/two-receivers setting (as in [2]), there is scope for cheap talk to generate additional surplus relative to the situation without information revelation.

Our experimental findings are that cheap talk does not generate any efficiency of entry and payoffs beyond the level that is obtained without information transmission. On average, senders’ (incumbents) messages convey truthful information. In case senders send deceptive messages, most of the time it concerns strategic deception (understating the state), but there is a robust substantial amount of obscure deceptions (overstating the state). The receivers’ (potential entrants) empirical probability to enter is increasing in the size of the market as it is claimed to be by the sender. Thus, despite cheap talk not being able to enhance overall performance, messages contain relevant information and this is picked up by the receivers. So, while we find overcommunication (relative to standard theoretical prediction) in the laboratory, compared to the situation without information transmission, cheap talk doesn’t work to alleviate the asymmetric information problem. The reason is that receivers fail to optimally translate the information received in their entry decision, possibly due to overcautiousness.

## Materials and Methods

In this section we present, subsequently, the central cheap talk setting, the treatments we implemented in our experiment and the corresponding hypotheses (based on standard theoretical prediction), and details on the precise design and procedures followed.

### Setting

**Fig 1:**
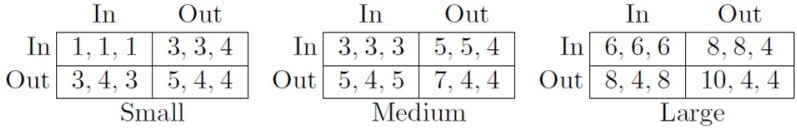
The signaling game with two strategically interacting receivers.

We consider a signaling game with one sender and two receivers. The game may be in one of three possible states of the world; each state being equally likely a priori. The sender is privately informed on the actual state and has the ability to inform the receivers on the actual state via one public message notifying both receivers about the state being drawn. The only information the receivers have about the true state is the message sent to them and they have to choose independently out of two alternative actions: In or Out. The action choices of both receivers together with the true state of the world, determines the payoffs of the three players. The resulting payoffs are displayed in Fig 1. In each cell, corresponding to the choices of the row receiver and the column receiver, the first entry presents the sender’s payoff, the second the row receiver’s, and the third the column receiver’s payoff. Notice that the payoffs do not depend on the message being sent; so, talk is cheap.

This setting is a natural analogue of an entry game (as in [11]) with one incumbent and two potential entrants and where the states of the world correspond to possible market sizes: small, medium or large. The potential entrants have no experience in the market that they consider entering, apart from the three states being equally likely, and nevertheless have to decide whether to enter or to stay out. By staying out, the potential entrant can get a sure reservation profit of 4. When entering, the resulting profit may be smaller or larger, depending on the true state and on the entry decision of the other potential entrant: the entrants’ (and so is the incumbent’s) profit is then given by 4 + 2(*c* − *m*), where *c* is the market capacity (with values 1.5, 2.5 and 4 in the small, medium and large market) and *m* is the number of firms active in the market (with 1, 2 and 3 being the possible values). In general, everything else equal, the potential profit of an actual entrant is larger if the market is larger in size and larger with less competitors in the market. This does not hold only for the potential entrants, but is also a structural characteristic of the incumbents profit. Therefore, the incumbent has an incentive to deceive the potential entrants by understating the state of the world when giving the opportunity to provide information on the market condition to them. Of course, the potential entrants are aware of this possibility of the preferences being misaligned in better market circumstances.

### Treatments and Hypotheses

Our primary treatment—which we dub the cheap talk (CT) treatment—is the setting described above. Apart from understanding how information is strategically transmitted in such a setting involving multiple strategically interacting receivers, we are interested in whether cheap talk can ameliorate part of the information disparity between the sender on the one hand and the receivers on the other hand. In order to make such a comparison, we have two baseline treatments: the full information (FI) treatment and the no information (NI) treatment. The latter baseline positions receivers in the worst situation where no information about the state is given; the former baseline places them in the ideal situation where they have complete information on the state.

In case the receivers are fully informed about the actual state of the worlds (treatment FI), both will stay out in the small state and will enter in the large state; in the medium state there are three equilibria: two where precisely one of the receivers enters and one in which each of them enters with fifty percent probability.

The case where the receivers have no information at all (treatment NI), possesses three equilibria: two pure asymmetric equilibria in which one of the receivers enters while the other stays out and one symmetric mixed equilibrium in which each of the receivers enters with two-thirds probability. Comparing these payoffs (see Table 2) with those in the FI treatment learns that all players involved prefer, from an ex ante perspective, the FI treatment over the NI treatment.

As in the cheap talk setting (treatment CT), the sender always attempts the receivers to believe that the true state is the small one, all messages loose their credibility. As a result, there are no separating equilibria. All “babbling equilibria” render the same outcomes as predicted for the NI treatment.

We can compare the performance of the CT treatment with the two baselines on the basis of two efficiency criteria: the first regards the frequency of efficient entries (that is, entry until excess profits are eroded), the second regards the payoffs generated. Table 1 summarizes the expected frequency of efficient entry levels in each of the states and the ex ante expected payoffs to the sender and receivers for each of the three treatments, as predicted by the symmetric equilibrium in the respective treatment.

**Table 1:** Predicted frequencies of entry levels and payoffs in the (symmetric) equilibria in the various treatments.

	Frequency of efficient entry levels				Expected payoff	
Treatment	Small	Medium	Large	Average	Sender	Receiver
Full info (FI)	1.00	0.50	1.00	0.83	5.33	4.67
No info (NI)	0.11	0.44	0.44	0.33	4.67	4.00
Cheap talk (CT)	0.11	0.44	0.44	0.33	4.67	4.00

On basis of the numbers in the table we can formulate the following two hypotheses:

**H.1-1.** The level of efficiency and the payoffs obtained in the CT treatment equals that of the NI treatment.
**H.1-2.** The level of efficiency and the payoffs obtained in the FI treatment exceeds that of the CT treatment and the NI treatment.

One reason to expect the first hypothesis to be rejected is that in cheap talk experiments overcommunication is a rather robust and frequently recurrent phenomenon (though, it is not clear that this phenomenon is robust to our extension to multiple strategically interacting receivers). That is, senders’ messages typically convey more (truthful) information than is predicted by sequential equilibrium prediction (assuming pure payoff-oriented and self-centered players), which is picked up by the receivers. If this is found to be true in the present setting, then cheap talk has the potential to increase the level efficient entry and payoffs relative to the NI treatment.

Our base hypotheses with respect to the information transmission and processing are formulated relative to the standard theoretical prediction (cf. [12]):

**H.2-1.** In the CT treatment, the frequency of chosen messages is constant over states.
**H.2-2.** In the CT treatment, the likelihood to enter is constant over messages.

The combination of these two hypotheses, provides us with a third testable hypothesis:

**H.2-3.** In the CT treatment, the likelihood to enter is constant over states.

Although it is to be expected that the latter hypothesis will only be rejected in case at least one of the former hypothesis is rejected, not being able to reject this hypothesis may go hand-in-hand with rejecting both of the former two hypotheses (but not with rejecting only one of them).

### Design and Procedures

For the two baseline treatments (NI and FI) we had in total five matching groups, while we ran the cheap talk treatment (CT) with eight matching groups. All matching groups consisted of twelve subjects, who played the game as specified by the treatment over a sequence of fifty rounds.

Every round again the subjects in a matching group were randomly partitioned into four triples and, for each triple, one member was assigned the sender role while the other two members got one of the receiver roles. Next, a state was drawn at random and the sender was informed on the selected state. The information the receivers received depended on the respective treatment. In the FI treatment, also the receivers were informed about the state. In the NI treatment, the receivers did not get any information. In the CT treatment, the only information receivers held was the message that was sent to them by the sender. To do so, the sender had three messages at her disposal that were of the type “the real state is …”. Given the information, next, the receivers had to choose whether to enter or to stay out. Depending on the choices of the receivers and the state drawn, the three members obtained their respective payoff.

The phrasing in the experiment was chosen as neutral as possible: the sender and receiver roles were named role A and role B, the states where presented as payoff Tables 1, 2 and 3, the messages were numbered similarly (“Table … has been selected”), and the actions were called option X and option Y. To facilitate learning, during the session, each subject had a history table displaying the table selected, her role, the decisions by the members in their triple, and her payoff. After the last round of play, three rounds were randomly drawn with replacement and subjects were paid according to the sum of the payoffs they made in the three rounds drawn. The rematching in triples within matching groups, the random role assignment and the lottery payment were implemented to mimic a one-shot interaction between subjects as much as possible.

**Table 2:** Frequency of efficient entry outcomes and payoffs in the various treatments.

	Frequency of efficient entry outcomes				Average payoff	
Treatment	Small	Medium	Large	Overall	Sender	Receiver
Full info (FI)	0.98	0.51	0.90	0.80	5.44	4.69
No info (NI)	0.26	0.48	0.22	0.32	5.29	4.11
Cheap talk (CT)	0.23	0.48	0.31	0.34	5.24	4.14

**Table 3:** Messages chosen by the senders in the different states.

	Message		
State	Small	Medium	Large
Small	0.69	0.22	0.10
Medium	0.50	0.41	0.10
Large	0.59	0.22	0.19
Overall	0.59	0.28	0.13

Subjects were invited and could sign up to participate in an economic experiment via ORSEE ([13]). The sessions were run in the BEElab at Maastricht University in May and June 2012. The instructions and comprehension questions were paper-based; the decision phase was computerized using z-Tree ([14]). In total 216 students participated in our sessions. An experimental session lasted between 60 and 90 minutes and the average earning of the subjects was 13.41 Euro, including a 3 Euro show-up fee. All instructions, software, data files (raw and processed) and codes used for analysis are retrievable from Figshare (doi: 10.6084/m9.figshare.3838020; url: https://figshare.com/s/d1f69bea9cbaf15d5c0d).

## Results

In total we have five independent observations for the baseline treatments (NI and FI) and eight for the treatment with cheap talk (CT). We do not find any substantial time trends in period averages for each matching group and we do not find much variation across matching groups for each cluster of ten periods. Therefore, we consider matching group averages over all periods in our data analysis.

### Treatment Comparisons

In this first subsection, we focus on the outcomes generated in the various treatments and the performance of the cheap talk treatment in particular. Thereby, we focus on the number of entrants in the various states and the average payoffs of the sender and the receiver(s).

**Fig 2:**
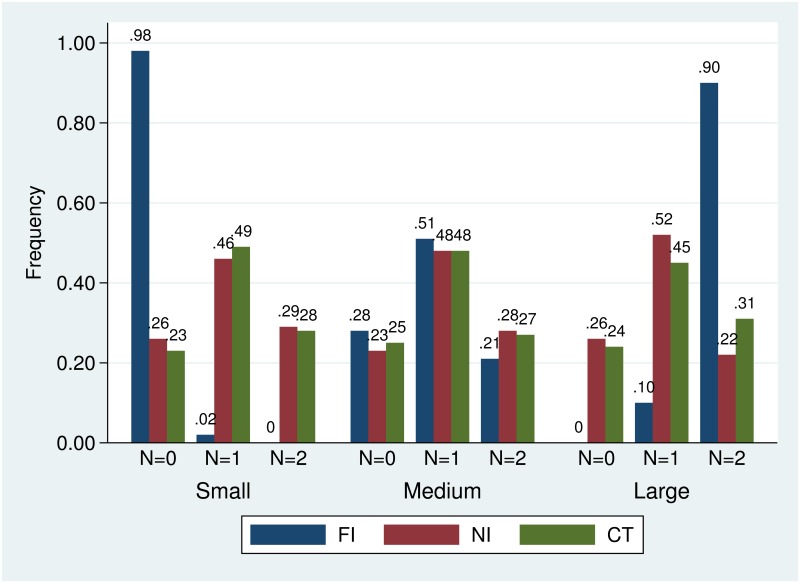
Frequency of the number of entrants (N).

Fig 2 presents the frequencies of the number of entrants in the different states for the three treatments. We see that in the FI treatment, there have been no instances where two receivers entered in the small state and where no receiver entered in the large state. From this we can conclude that the structure of the payoffs has been clear to all participants. Furthermore, we see that the differences between the NI treatment and the CT treatment are rather small. Only in the large state there is a small difference visible; here, the expected number of entrants is 0.96 in the NI treatment and 1.07 in the CT treatment. Where cheap talk communication appears to successfully support entry in the large state, it fails to prevent receivers from entering in the small state. Yet another striking observation is that the entry outcomes are quite similar across all treatments in the medium state. While this is close to symmetric equilibrium prediction for the FT treatment, for the NI treatment and the CT treatment there is substantially less entry compared to symmetric equilibrium prediction.

Next, we compare the performance of the three treatments on basis of two efficiency criteria. The first efficiency criterium relates to the frequency of efficient entry levels in the different states. In a monopolistic competitive market, firms usually enter up to the moment that all excess profits from entry are eroded. In our configuration, this would mean that (if all information were to be available) there should be no entry in the small state, one entrant in the medium state and two entrants in the large state. Table 2 shows for each treatment the frequency of efficient entry outcomes per state and on average over states.

The frequencies of efficient entry levels for the FI treatment are quite in line with the theoretical predictions in Table 1. For the other two treatments the frequency of efficient outcomes is larger in comparison to theoretical prediction when the state is small or medium, but lower in case the state is a large. These opposite effects lead to the overall frequency of efficient entry levels not being substantially different as predicted by theory.

On the overall level, the frequency of efficient entry outcomes in the CT treatment does not differ from that in the NI treatment (Mann-Whitney, two-sided: *p* = .5549). With regard to these frequencies on state-level, there are no significant differences between these two treatments in the small state (*p* = .2403) and the medium state (*p* = .2403). However, and as was already visible in Fig 2, cheap talk communication induces more efficient entry in the larger state (*p* = .0475). But, this effect is not sufficient to make cheap talk communication effective on the overall level.

Comparing these two treatments with the FI treatment, there is no difference in the medium state (CT: *p* = .4624; NI: *p* = .6004). However, in the small state and large state, the frequency of efficient entry outcomes in the FI treatment significantly exceeds that of the NI and CT treatment (all tests: *p* < .01). These differences are sufficiently strong to make the FI treatment more efficient than the other treatments on the overall level (both test: *p* < .01).

With respect to the expected payoffs, our findings are comparable to those for the frequencies: There is no significant difference between expected payoffs in the CT treatment and the NI treatment (sender: *p* = .7140; receivers: *p* = .5582) and the expected payoffs in the CT treatment are below that in the FI treatment (sender: *p* = .0669; receivers: *p* = .0034).

**Result 1-1.** Cheap talk communication fails to increase efficiency and payoffs beyond the level that is obtained in a setting where receivers do not receive any information on the actual state.
**Result 1-2.** The level of efficiency and payoff that is obtained in a setting where receivers are perfectly informed about the state cannot be achieved when receivers are informed via messages sent by the sender.

In order to better understand why cheap talk communication failed to enhance efficiency relative to the situation where receivers do not obtain any information regarding the state in our experiments, we continue with an analysis of the senders’ and receivers’ behavior in the cheap talk treatment.

### Individual Behavior

Table 3 shows the probabilities by which the senders have chosen messages in the different states. One property that stands out is that in each column the number on the principal diagonal exceeds the two numbers off the diagonal. Since the signaling behavior of the sender does not differ much across matching groups, it does not come as a surprise that we can reject the hypothesis that probabilities by which messages are chosen are independent of the state: Given a message, the probability by which this message is chosen is significantly higher in the state that matches the message compared to the other two states (Wilcoxon, two-sided, *p* < .03 for all six comparisons) and are not significantly different between the other two states (*p* > .12 for all three comparisons).

**Result 2-1.** The messages sent by the sender do convey information on the actual state.

The dominance of the diagonal in the matrix that reflects signaling behavior in the cheap talk treatment, indicates that on the aggregate level senders tell the truth excessively compared to standard theoretical prediction. So, the excessive truth-telling that has been found in earlier literature (cf. [8, 9]) appears robust to introducing multiple strategically interacting receivers. The numbers in the table also indicate a general tendency for strategic deception (understating the state) and a substantial amount of obscure deception (overstating the state). We will provide a further discussion on these two forms of deception in the discussion.

In order to estimate the amount of truth-telling, we decompose the sender’s signaling behavior into a truth-telling component and a state-independent component:

$$\left(\begin{array}{ccc} m_{S,S} & m_{S,M} & m_{S,L}\\ m_{M,S} & m_{M,M} & m_{M,L}\\ m_{L,S} & m_{L,M} & m_{L,L} \end{array}\right)=\lambda \left(\begin{array}{ccc} 1 & 0 & 0\\ 0 & 1 & 0\\ 0 & 0 & 1 \end{array}\right)+\left(1-\lambda \right)\;\left(\begin{array}{ccc} \sigma_{S} & \sigma_{M} & \sigma_{L}\\ \sigma_{S} & \sigma_{M} & \sigma_{L}\\ \sigma_{S} & \sigma_{M} & \sigma_{L} \end{array}\right)$$

and estimate the four parameters that fit our data best:

$$\lambda =0.1365\;\sigma_{S}=0.62\;\sigma_{M}=0.27\;\sigma_{L}=0.10.$$

These numbers indicate that if we were to partition the population into truth-tellers and perfect babblers, we would estimate about 14% of the population to consist of truth-tellers. Moreover, we see that although any state-independent signaling behavior can be supported as a babbling equilibrium, the perfect babblers have a general tendency to understate the state. These understatements may be a strategic response to the presence of truth-tellers and receivers showing moderate trust in messages.

Now that we know that messages do convey information, the next question is whether this is picked up by the receivers. Table 4 presents the entry decisions of the receivers in response to messages received. We see that the probability by which the receivers enter are increasing in the message, and find that the differences are significant (S-M: *p* = .0251; M-L: *p* = .0117). This result implies that the excess truth-telling is picked up by the receivers and their responses reflect some trust in the messages.

**Table 4:** Entry decisions of the receiver after the different messages.

	Entry decision	
Message	In	Out
Small	0.45	0.55
Medium	0.55	0.45
Large	0.81	0.19
Overall	0.53	0.47

**Result 2-2.** Receivers respond to messages in a way that reflects that they regard messages somewhat trustworthy.

Table 5 combines the information presented in the previous two tables and shows the probability that the receivers enter for each state.

**Table 5:** Entry decisions of the receiver in the different states.

	Entry decision	
State	In	Out
Small	0.51	0.49
Medium	0.53	0.47
Large	0.54	0.46
Overall	0.53	0.47

We see that the receivers’ entry probabilities for the three states are quite close. Yet, the probability by which receivers enter is significantly lower in the small state compared to the other two states and there is no significant difference between the larger two states (S-M: *p* = .0357; S-L: *p* = .0173; M-L: *p* = .2626).

**Result 2-3.** Cheap talk communication results in minor differences in entry decisions across states.

In sum, we find that messages are informative and this has been picked up by the receivers, but that the receivers’ entry decisions in response to the messages is such that it does not produce sufficient differences in entry probabilities across states for cheap talk to be able to enhance efficiency.

## Discussion

In this section we first aim to provide an explanation for why, despite senders’ messages conveying information and the receivers recognizing this and responding to that, cheap talk does not induce an enhancement of efficiency levels and payoffs beyond that obtained without any information transmission. Second, we try to increase understanding in the senders’ substantive use of messages that are destructive for the payoffs of all parties involved by investigating several alternative underlying motives that may play a role.

### Possible Explanation

Table 6 shows the entry probabilities for the receivers in the symmetric equilibrium, given the sender’s behavior in the experiment. Comparing these numbers with those in Table 4, we see that, while receivers do realize that the senders’ messages capture some amount of truthful information, they fail to respond optimally. Receivers simply behave overly cautious after every possible message (which contrasts the finding by [15] where subjects excessively enter into competition, which they attribute to overconfidence). This cautiousness was something that was also observed in the NI treatment, where receivers only enter with 51% probability, while equilibrium predicts them to enter with 67% probability.

**Table 6:** Equilibrium behavior of the receivers given the sender’s behavior.

	Entry decision	
Message	In	Out
Small	0.61	0.39
Medium	0.64	0.36
Large	1.00	0.00
Overall	0.67	0.33

One obvious candidate explanation for the overly cautiousness to enter is risk aversion. In the FI treatment the receivers enter with 47% probability in the medium state, which is close to the symmetric equilibrium prediction. However, in the CT treatment the receivers enter with overall 53% probability which is far below the 64% that equilibrium predicts given the sender’s behavior (Table 6). In the post-experimental questionnaire we elicited our participants’ risk attitudes by the direct approach as suggested in [16]. That is, we asked the participants the question “How do you see yourself: Are you generally a person who is fully prepared to take risks or do you try to avoid taking risks?”. Next they were asked to tick a box on a scale from 0 to 10 where the value 0 means: “not at all willing to take risks” and the value 10 means “very willing to take risks”. We find that participants in the CT treatment do not report a different risk attitude compared to those in the FI treatment (Mann-Whitney, two-sided, *p* = .8103) with average responses of 5.96 and 6.06 respectively. Hence, the relative low entry rate in the CT treatment is not due to individuals in this treatment differing in risk attitude. So, in order for risk attitude to be a driver of this difference, it has to be the difference in the nature of the risk between the two treatments. Notice here that while the strategic uncertainty concerning the other receiver’s entry decision in both treatments are comparable, in contrast to the FI treatment there is also uncertainty regarding the actual state being drawn in the CT treatment.

### Obscure Deception

Despite there being excess truth-telling on the aggregate level, only in 43% of the cases senders told the truth. Obviously, our setting invites senders to deceive the receivers by understating the true state and it is not too surprising to find this to happen. However, in about one-fourth of the instances where a sender sent a deceptive message, she actually overstates the state—and this number is stable over time and across matching groups. Where there are clear strategic motives for understating the state (strategic deception), these overstatements appear somehow a bit obscure (obscure deception). Still, about three-fourth of the subjects at least once sent such an obscure deceptive message and about one-fourth of the subjects sent such a message in at least 20% of the instances they acted as sender. Given the large number of individuals using such a message and the use not vanishing over time, we further investigate on possible motives for using such a message.

Table 7 shows the costs and gains from lying for the sender and receiver, given the actual response to the messages by the receivers in the experiment. The first number in each cell corresponds to the gain to the sender of sending the respective message in the respective state; the second number corresponds to the gain for the receivers. We see that it is always beneficial for the sender to understate the actual state. Where these understating lies are harmful for the receivers in the large state, they are beneficial for them in the medium state. The reason for this is that the receivers enter with 55% probability (recall Table 4) after receiving the message saying that the state is medium, which is too high in the medium state. Sending the message saying that the state is small in this state helps the receivers in suppressing the entry probability to 45%, which happens to be beneficial for the receivers.

**Table 7:** Costs and gains from lying.

	Message		
State	Small	Medium	Large
Small	—	−0.42, −0.32	−1.44, −1.26
Medium	+0.42, +0.11	—	−1.01, −0.44
Large	+1.44, −0.53	+1.01, −0.32	—

Overstating the actual state is always harmful for the sender and the receivers. It is questionable why in 32% of the instances in the small state and in 10% of the instances in the medium state such messages have been chosen by the senders. A strategic motive may be to spoil the communication channel in order to implement a babbling equilibrium. However, since the interaction properties in our experiment mimic that of a one-shot interaction (co-player rematching, re-assignment of roles, anonymous decision making, not all rounds are selected for payment, and in addition there is variation in states being selected), it is hard to build a babbler reputation. Therefore, this strategic motive seems not to be a plausible explanation for the high amount of obscure deceptions.

One other experimental study in which nonstrategic deception has been found is [17]. That study considers a version of the trust game where the investor is not aware of the possible benefit on investment. The investee, who is aware of this benefit, has the capacity to transmit information on it to the investor prior to his investment decision. The investee has a clear strategic motive to overstate the benefit in order to persuade the investor to invest a lot. Although more instances are found in which investees overstate the benefit, there are a substantial amount of instances in which they understate the true benefit. Their explanation for these nonstrategic deceptions is guilt aversion: by understating the benefit, investees decrease the return expectations of the investors, thereby decreasing their guilt from a possibly disappointing return. As in our situation the sender of the message has no further actions to take, guilt considerations seem not to play any role.

Another emotional motive for sending such a lie, that is given in [18], is a “spiteful reaction to unfair behavior”. Therefore these lies are classified “spiteful black lies” in [19] (which is very close to what we call obscure deceptions with the main difference being that in our case the negative consequences of these lies is according to payoff expectations based on the experimental data). As participants in our experiment have been rematched and reassigned their role every round anew, such a spiteful reaction typically targets someone that cannot be held accountable for having caused a feeling of being treated unfairly. Nevertheless the spiteful reaction may be a mere act of getting rid of such negative feelings. Two prominent experiences that we recognize as potential triggers for spiteful response are: (1) having suffered from a lie as a receiver, and (2) having suffered from not having been trusted as a sender after having told the truth. Out of the 202 instances of obscure deceptions from the second round onwards, (1) only thirteen (6.44%) were sent after having received a payoff lower than the reservation payoff of 4 after a lie has been told to them as a receiver in the previous round, and (2) only eighteen (8.91%) were sent after having received a payoff below the full coordination payoff of 5 after having told the truth in the small or medium state as a sender in the previous round. From these numbers we infer that the high amount of obscure deceptions is not likely to be explained by spiteful reactions to unfair behavior.

Yet another explanation of these obscure deceptions may be found in “self-image” considerations. The idea is that a sender who recognizes her incentive to understate the state and that the receivers may be aware of this, may expect that the receivers expect her to do so. By overstating the state, she can avoid being considered a “selfish liar”, thereby distinguishing herself from others who belong to this group. One other way to avoid being labeled as such is of course to tell the truth. Still, in the small state telling the truth does not help to get rid of a possibly “selfish” image. These arguments are valid even in the one-shot interaction that our design aims to mimic. In our experimental data, we do not see any evidence that goes against self-image concerns being a plausible motive for the obscure deceptions in our experimental population. Although 74% of our experimental population has been responsible for at least one obscure lie, about one-thirds of the population has been responsible for 70% of the obscure lies and 19% of the population committed an obscure lie in more than one-fourth of the instances (while in the large state it was impossible to commit such a lie).

## Conclusion

In this paper we investigate in a setting with strategic tensions between a sender (incumbent) and two strategically interacting receivers (potential entrants) whether cheap talk can enhance efficiency (in terms of efficient entry levels and payoffs). Although there is excessive truth telling and this is picked up by the receivers, we do not find cheap talk to enhance efficiency. The reason for this is that receivers fail to optimally translate the information received into their entry decision. As illustrated by the examples of traffic congestion and dating platform, the interaction among uninformed receivers makes cheap talk more complicated compared to the situation in which receivers do not strategically interact. When the interests of sender and receivers are aligned, cheap talk may improve efficiency but through an untruthful message. When the sender has a conflict of interest with the receivers, cheap talk may be still informative to some degree due to the overcommunication phenomenon but it is not enough to ameliorate the efficiency. An implication of this study is that we cannot rely on cheap talk to reduce the inefficiency caused by informational asymmetry in a complicated strategic environment.
